# Hybrid Transthoracic Periventricular Device Closure of Ventricular Septal Defects: Single-Center Experience

**DOI:** 10.21470/1678-9741-2020-0115

**Published:** 2021

**Authors:** Liying Wu, Ibrahim Cansaran Tanidir, DongTing Ye, Xiong Zhang, Bin Li, Daliang Zhu, Gaopi Deng, Haisheng Chen

**Affiliations:** 1 Department of Cardiovascular Surgery, Guangzhou First People’s Hospital, School of Medicine, South China University of Technology, Guangzhou, Guangdong, China.; 2 The First Clinical Medical College, Guangzhou University of Chinese Medicine, Guangzhou, China.; 3 Department of Pediatric Cardiology, Istanbul Saglik Bilimleri University Istanbul Mehmet Akif Ersoy Thoracic and Cardiovascular Surgery Education and Research Hospital, Istanbul, Turkey.; 4 Department of Gynecology, The First Affiliated Hospital of Guangzhou University of Chinese Medicine, Guangzhou, China.

**Keywords:** Heart Septal Defects, Ventricular, Bundle-Branch Block, Mitral Valve Insufficiency, Tricuspid Valve, Aortic Valve Insufficiency, Echocardiography, Cardiac Surgical Procedures

## Abstract

**Objective:**

To evaluate the efficacy of hybrid transthoracic periventricular device closure of ventricular septal defects (VSDs) in a single center.

**Methods:**

All patients who underwent hybrid transthoracic periventricular device closure of VSDs between January 2018 and December 2019 were retrospectively analyzed. The preoperative, operative and postoperative findings and clinical follow-ups were reviewed.

**Results:**

A total of 59 patients underwent the procedure. Transesophageal echocardiographic guidance was used in all procedures. The procedure was successful in 57 procedures (97%). The procedures of two patients were changed to open-heart surgery during the same intervention due to severe aortic insufficiency (the device was not deployed) and significant residual shunt after device deployment. One major complication (1.7%) was observed after the procedure. The patient’s device was dislodged within 12 hours after the procedure, and this patient underwent device extraction and VSD patch closure due to significant residual shunt. Eight (14%) minor complications were observed after the procedure, and three of them persisted during follow-up. Three of these eight complications were incomplete right bundle branch block, one of which resolved during follow-up; two were mild residual shunts, one of which resolved during follow-up; two were mild new-onset tricuspid valve insufficiencies; and one was mild new-onset mitral valve insufficiency; all valvular insufficiencies were resolved during follow-up.

**Conclusions:**

Hybrid transthoracic periventricular device closure of VSD seems to be a good alternative approach due to its procedural success and low risk rates. The best advantage of the procedure is the possibility of switching to open-heart surgery, if necessary.

**Table:** 

Abbreviations, acronyms & symbols				
**AI**	**= Aortic insufficiency**		**MI**	**= Mitral insufficiency**
**ASD**	**= Atrial septal defect**		**PDA**	**= Patent ductus arteriosus**
**cAVB**	**= Complete atrioventricular block**		**pmVSD**	**= Perimembranous ventricular septal defect**
**CI**	**= Confidence interval**		**RV**	**= Right ventricle**
**CPB**	**= Cardiopulmonary bypass**		**SPSS**	**= Statistical Package for the Social Sciences**
**CHD**	**= Congenital heart defects**		**TEE**	**= Transesophageal echocardiography**
**ECG**	**= Electrocardiography**		**TI**	**= Tricuspid insufficiency**
**iRBBB**	**= Incomplete right bundle branch block**		**TTE**	**= Transthoracic echocardiography**
**LV**	**= Left ventricle**		**VSD**	**= Ventricular septal defect**

## INTRODUCTION

Minimally invasive surgery for congenital heart disease in pediatric/adult patients traditionally refers to therapeutic approaches designed specifically to minimize the physical trauma associated with surgery and thereby maximize the likelihood of rapid recovery with minimal morbidity. Many of these approaches are possible due to technological innovation^[1]^.

Ventricular septal defects (VSDs) are one of the most common congenital heart defects (CHDs), accounting for 20-30% of all forms of congenital cardiac malformations, and 80% of VSD cases are perimembranous VSDs (pmVSDs). Conventional surgical repair of VSDs via cardiopulmonary bypass (CPB) is the gold standard. However, this approach cannot avoid the potential for CPB-related complications, complete atrioventricular block (cAVB), surgical incision scars, or prolonged recovery. With the improvement in development of various devices, the transcatheter device closure of pmVSDs has also gradually gained popularity in most medical centers with a promising closure success rate. Since the 1990s, innovation in medical devices, multimodality imaging and operative instruments, as well as the strong desire of patients and their parents for less invasive surgical procedures, has led to the development of minimally invasive surgery for congenital heart disease. Based on the two methods mentioned (surgical and transcatheter), the hybrid periventricular device closure of VSDs was designed to combine the advantages of both approaches, allowing direct access to the defect without CPB but with guidance by transesophageal echocardiography (TEE) or transthoracic echocardiography (TTE). These procedures, which involve smaller incisions, are designed to produce better cosmetic results and reduce rehabilitation time and pain compared to traditional open-heart surgery. This method has been widely used in China with promising results^[1-9]^. 

Herein, we report our experience with hybrid transthoracic periventricular device closure of VSDs.

## METHODS

### Patient Selection

Patients who underwent hybrid transthoracic perventricular device closure of VSDs between January 2018 and December 2019 at Guangzhou First People’s Hospital in China were retrospectively analyzed. The study protocol was approved by the Institutional Ethics Committee of Guangzhou First Municipal People’s Hospital.

The initial evaluation of VSDs was performed by the same surgeon (LW) with TTE. Patients in whom TTE showed a suitable morphology, size, and location for device closure according to the guidelines^[10]^ were selected for the procedure. Periventricular device closure is the first treatment of choice in our center. We claim that the ideal perimembranous defect for device closure should have at least 2 mm aortic rim and a single exit point on the right ventricular side. In addition, due to the fact that the selected device will be 1-2 mm larger than the defect, we do not prefer to close defects larger than 12 mm (either right ventricular side or left ventricular side). The remaining patients underwent surgical closure.

### Device and Device Selection Criteria

The VSD occluder was a double-disc, self-expandable symmetric device produced by Shenzhen Lifetech Scientific Ltd. Lifetech Cera™ membrane VSD occluder devices are classified as symmetric, eccentric, or asymmetric. In our patients, we used symmetric Cera™ membrane VSD occluder devices. The diameter of the device was selected to be 1-2 mm larger than the diameter of the defect. 

### Procedure

**Fig. 1:**
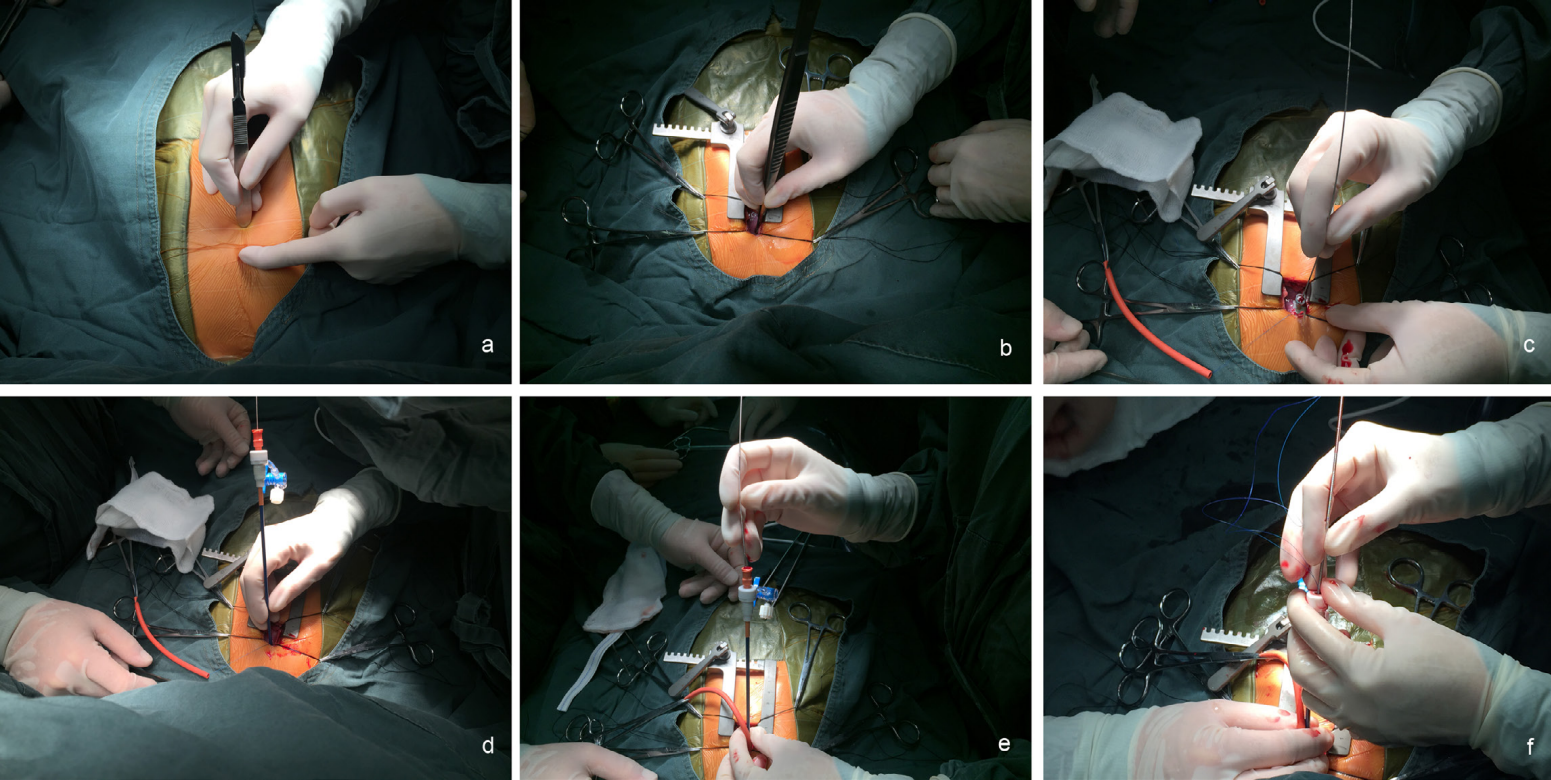
Images of the procedure. A: planning the incision site. B: selection of the puncture site in the right ventricle. C: an 18G venous indwelling needle was inserted through a purse-string suture, and a guidewire was advanced to the right ventricle. D: the needle was removed, and a delivery sheath was advanced over the guidewire. E: the device was advanced through the delivery sheath. F: the device was deployed after the device was in the correct position.

**Fig. 2:**
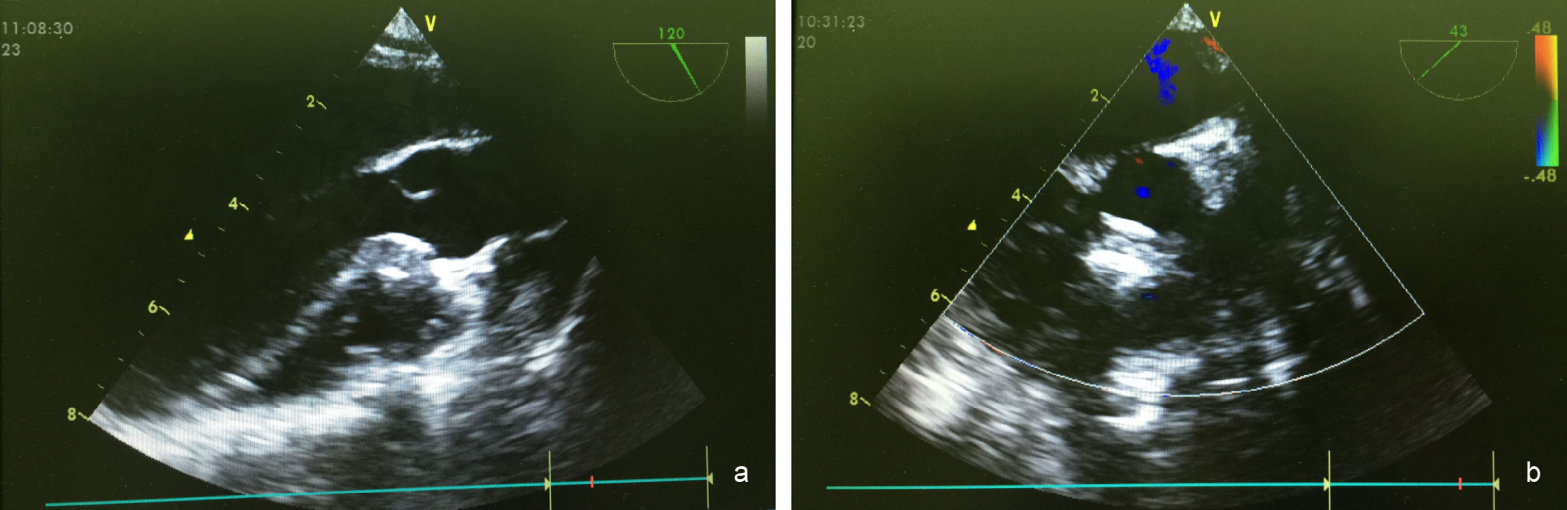
Transesophageal echocardiography after the procedure. A: long axis 2D view of the device. B: short axis of the device with color Doppler.

A GE Vivid 7 6T/9T multiplane probe (General Electric Company) was used to determine the accurate size and morphological features of the VSD, visualize the rims of the VSD, and guide the procedure in all cases. After induction of general anesthesia and intubation, the morphology, location and size of the defect and valvular functions were evaluated with TEE. After a lower partial median sternotomy incision (2 cm) was made (Figure 1A), the pericardium was opened and suspended to expose the anterior free wall of the right ventricle (RV). Systemic heparinization was performed with 100 U/kg of unfractionated heparin. The puncture sites on the RV were selected perpendicular to the defect, located under the guidance of TEE and free from the right coronary artery (Figure 1B). A purse-string suture was placed around the puncture site. An 18G venous indwelling needle was inserted through a purse-string suture, and then a guidewire was advanced through the exit hole of the needle into the RV (Figure 1C). The needle was removed, and a delivery sheath was advanced over the guidewire into the RV (Figure 1D). The guidewire was advanced through the defect into the left ventricle (LV) under the guidance of the TEE. Then, the delivery sheath was advanced into the LV over the guidewire, and the inner sheath of the delivery sheath and guidewire were removed. After preparing the occluder device according to the user’s guide, it was advanced through the sheath (Figure 1E). The left-sided disc was opened both by retracting the sheath and advancing the cable in the LV and by retracting the waist, then the whole system was pulled back into the defect and the right-sided disc was deployed into the defect. A push-pull test was performed to evaluate the stability of the device. Before releasing the device, any residual shunt or new-onset tricuspid, mitral or aortic valve obstruction or insufficiency was evaluated with TEE. If no problems were observed, the device was released (Figure 1F). Afterward, the device position and residual leaks were again evaluated with TEE (Figure 2). Then, both the delivery sheath and the cable were withdrawn, and the purse-string suture was tied. The pericardium was reapproximated, and a drainage tube was placed. The incision was closed in layers, as described in detail in the literature^[4,8]^. 

### Additional procedure

In two patients with patent ductus arteriosus (PDA), surgical ligation was used before the periventricular VSD device closure. In a patient with atrial septal defect (ASD), an ASD closure device guided by TEE through a 2-cm incision on the right margin of the sternum in the fourth intercostal space was used for closure. 

### Follow-Up

Prophylactic antibiotics were started before the procedure and continued for 2 days. During the first 24 hours after the procedure, continuous electrocardiography (ECG) monitoring was used to detect arrhythmias. Echocardiography was performed to monitor acute complications and residual shunts immediately and 24 hours after the procedure. Most patients were discharged 3-5 days after the procedure and were maintained on aspirin (3-5 mg/kg/day) for 6 months. 

Routine follow-up examinations included physical examinations, ECG, and echocardiography and occurred at 4-6 weeks, 3 months, 6 months, 12 months, and annually thereafter.

### Statistical Analysis

Statistical analyses were performed with SPSS Statistics 23.0 (IBM Corp., Armonk, NY, USA). Continuous variables are expressed as mean ± standard deviation or median. Categorical data are presented as frequencies and percentages. 

## RESULTS

### Demographics

Detailed demographic characteristics of the patients are provided in Table 1.

**Table 1:** Baseline characteristics.

Age at operation (years, median, range)		3.0	(range, 5 months-40 years)
Weight (kg, median, range)		13	(range, 5-70 kg)
Female/Male, n (%)		33/26	(56%/44%)
Echocardiography	TTE VSD LV size (mm, mean±SD)	5.1±2.1	(range, 2.5-14 mm)
	TTE VSD RV size (mm, mean±SD)	4.5±1.5	(range, 2.1-8.8 mm)
	TEE VSD LV size (mm, mean±SD)	5.4±2.2	(range, 2.5-13 mm)
	TEE VSD RV size (mm, mean±SD)	4.7±1.7	(range, 2.4-10 mm)
Defect position, n (%)	Perimembranous	58/59	98%
	Muscular	jan/59	2%
	Aneurysm, n (%)	nov/58	19%

TEE=transesophageal echocardiography; TTE=transthoracic echocardiography; VSD=ventricular septal defect

### Procedural and Early Postprocedural Data

Success: procedural success was achieved in 57 of 59 procedures (96.6%). One patient had severe aortic valve insufficiency (AI), detected when the occluder was placed into the defect. Although repositioning of the device was attempted, AI did not recover. The device did not deploy, and the VSD was closed with a patch via median sternotomy. The other patient had a significant residual shunt after device deployment. This patient underwent surgical device extraction and VSD patch closure.

Residual shunt: a total of three patients had residual shunt after the procedure. One of these shunts was significant due to device dislodgement, and the patient underwent surgical closure the next day. The other two shunts were clinically insignificant.

Other interventions: in two patients and one patient, respectively, PDA and ASD were closed during the same procedure. Detailed information is provided in Table 2.

**Table 2:** Procedure-related data.

	n	%	Literature***
Procedure time (min, mean±SD, range)	104±31(45-186)		
Echocardiography, n (%)			
Transesophageal	59/59	100%	
Success	57/59	96.6%	92%
			(95% CI: 0.90-0.94)
Device sizes, n (%)			
5	7/57	12%	
6	15/57	26%	
7	17/57	30%	
8	10/57	18%	
10	5/57	9%	
12	3/57	5%	
Converting to surgical repair	2/59	3.4%	80/1368
			(5.8%)
Clinically insignificant residual shunt	2/59	3.4%	95/1368
			(6.9%)
Total severe complications	3/59	5.3%	109/1368
			-8%
Intraoperative complications	2/59	3.4%	88/1368
			-6%
			(95% CI: 0.028-0.071)
Significant residual shunt*	1/59	1.7%	32/1368
New-onset aortic valve insufficiency*	1/59	1.7%	31/1368
Complete heart block	None		10/1368
Failure to establish a path	None		8/1368
New-onset tricuspid valve insufficiency	None		7/1368
Postoperative early complications	1/57	1.7%	12/1368
			0.9%
Significant residual shunt	None		0
New-onset aortic valve insufficiency	None		0
Complete heart block	None		5/1368
New-onset tricuspid valve insufficiency	None		1/1368
New-onset tricuspid valve insufficiency	None		
Occluder dislodgement**	1/57	1.7%	2/1368
Second operation**	1/57	1.7%	4/1368
Follow-up period	No major complications	0%	9/1368

iRBBB=incomplete right bundle branch block.

* The patient's device was extracted and surgical closure was performed.

** Same patient.

*** Hong et al.^[5]^.

### Complications and Intensive Care Follow-Up

The total complication rate was 9/57 (15.8%). The major complication rate was 1/57 (1.7%), and the minor complication rate was 8/57 (14%). The main complication was device dislodgement, which occurred 12 hours after the operation. The device position changed, but it was not embolized. Due to the significant residual shunt, this patient underwent device removal and surgical VSD closure the next day. One patient had both incomplete right bundle branch block (iRBBB) and mild residual shunt with her VSD.

The mean extubation time was 5±2.6 hours (range, 1-16 hours), and the mean length of stay in intensive care was 22±10 hours (range, 5-63 hours). The mean chest tube removal time was 1.6±0.7 days (range, 1-3 days), and the mean length of hospital stay was 9±4 days (range, 4-20 days). 

### Follow-Up Data

All patients were followed for a period of 3-24 months (median, 10 months) with TTE and ECG: 31 patients for 3 months, 31 patients for 6 months, 26 patients for 1 year and 2 patients for 2 years. The follow-up rate was 100%. Detailed information is provided in Table 3.

**Table 3:** Follow-up data.

	n	%	Literature*
Follow-up (days, mean±SD, range)	57/57		
	(345±193)		
Residual shunt during follow-up			1/1368
			0.1%
Mild	2/57	3.4%	
Severe			1/1368
New-onset mitral valve insufficiency during follow-up			0
Mild	1/57	1.7%	
New-onset tricuspid valve insufficiency during follow-up			
Mild	2/57	3.4%	
New-onset aortic valve insufficiency during follow-up	None		5/1368
			0.5%
			(95% CI: 0.000-0.000)
Arrhythmia			
iRBBB	2/57	3.4%	11/393
			2.8%**
Permanent complete heart block	None	0%	3/1368

iRBBB=incomplete right bundle branch block

* Hong et al.^[5]^.

** Xing et al.^[7]^.

Valvular insufficiency:

Preexisting valvular insufficiencies: 

A proportion of 3/57 patients had mild AI before the operation. One patient’s AI regressed during follow-up, and two of these patients still had mild AI. However, there were no new-onset AI cases after the procedure. A proportion of 28/57 patients had mitral valve insufficiency (MI) before the operation, and 12 patients’ MI regressed during follow-up. However, 16 of these patients still had mild MI. 

New-onset valvular insufficiencies:

There were a total of three cases of new-onset valvular insufficiency after the procedures. 

There were two cases of new-onset tricuspid valve insufficiency after the procedure and diminished during follow-up. 

One patient had new-onset mild MI after the procedure, which resolved during follow-up.

ECG: there were no cases of cAVB after the operation or during follow-up. The only change on the ECG was iRBBB in 3/57 patients. During follow-up, one of the patient’s iRBBB resolved, whereas two patients still had iRBBB, which is clinically insignificant. 

Residual shunt: only two patients had residual shunt after the procedure. Residual flows from both patients were less than 1.5 mm, and one patient had muscular VSD. Both patients were followed up without any medications. 

## DISCUSSION

Periventricular device closure is a common treatment for VSD, especially in China. The first real off-pump periventricular device closure of a VSD was conducted in animal experiments in 1997 under TEE guidance and then applied in an infant with muscular VSD. Subsequently, periventricular device closure of a pmVSD was first reported in 2004. Recently, this technology has been widely used in China^[5,6]^.

Hybrid periventricular device closure of VSDs offers several advantages:

It can be used for any patient, regardless of age or weight.

CPB and its related complications (less bleeding, surgical and psychological trauma, cosmetic concern with scar formation, etc.) can be avoided.

The procedure is minimally invasive and rapid.

The risk of intimal injury by interventional catheterization can be avoided.

Due to the short sheath, route complications (sheath manipulation, device position adjustment, reliability to test the device stability, and long sheath damage to other tissues) and manipulations can be improved.

The procedure and its effectiveness can be monitored by TEE or TTE, which prevents the adverse effects of contrast agents and X-ray imaging and provides the ability to instanty evaluate the relationship of the device and valves.

If closure cannot be achieved or any complication occur, a rapid transition to open-heart surgery is possible^[2,3,5,7,8,11-13]^. 

### Procedural Success

The pooled estimate of the overall success rate of device closure in the 15 studies was 0.95 (95% CI: 0.92-0.97), and if the three studies with a 100% success rate were excluded, the pooled success rate was 0.92 (95% CI: 0.90-0.94, *I*^2^ = 31.1%, *P*=0.142)^[5]^. In addition, different studies report a higher success rate than for both transcatheter and periventricular device closure for pmVSDs by more than 93%^[11,14]^. Our success rate was 97%, which is similar to the rates in the literature. This finding shows that the procedure could be performed by surgeons with high success rates. Our unsuccessful cases occurred due to AI before deployment of the device and significant residual flow after deployment of the device. In addition, these patients were treated with a conventional surgical technique. Therefore, the VSD closure success rate was 100%, but the hybrid approach success rate was 97%. If any complications occur during the procedure, physicians can immediately convert the procedure to open-heart surgery and VSD patch closure. This option is the main advantage of this type of procedure. 

### Complications

It has been reported that intraoperative complications (up to 10%) immediately resolve after removal of the device. Therefore, the selection of an occluder of suitable type and size has been claimed to be the most crucial factor for complications^[5,6]^. Hong reported a severe complication rate of 109/1368 (8%) (88/1368 (6%) intraoperatively, 12/1368 (0.9%) in the early postoperative period, and 9/1368 during follow-up)^[5]^. Our severe complication rate was 3/59 (5.3%) (2 intraoperative and 1 postoperative), which is similar to the rates in the literature.

Residual shunt: severe residual shunt after the procedure was extremely low. This finding was due to the echocardiographic evaluation during the procedure. The most common minor complication was residual shunt, which was documented in 95 patients among 1368 patients in 15 studies. The pooled rate of postoperative residual shunt was 0.02 (95% CI: 0.01-0.03, *I*^2^=87.3%, *P*<0.001), and the pooled rate of follow-up residual shunt was 0.001 (95% CI: -0.001-0.002, *I*^2^=30.5%, *P*=0.126)^[5]^. In our series, there were 2 patients (3.4%) with residual shunt, which is slightly more than the number of patients with residual shunt in the literature. Both instances of shunt were mild and, during follow-up, these residual shunts will likely disappear, as suggested in the literature^[2]^.

Valvular problems: general valvular problems are AI, tricuspid insufficiency (TI), or MI. However, tricuspid stenosis (due to entrapment of the anterior leaflet chordae) has also been reported^[15]^. Some centers’ eccentric devices are used to decrease AI^[7]^. Xing Q et al.^[7]^ reported a 0.5% incidence of AI. In a series by Zhou et al.^[16]^, 6/41 patients had new-onset TI. The authors concluded that trivial TI can be accepted because it may decrease or disappear due to improvements in compression of the septal leaflet or chordae tendineae in the beating heart over time. In our series, there was no AI. However, two patients had mild TI, and one patient had mild MI, which was clinically insignificant. All of these complications were resolved during follow-up. However, one of our patients had significant AI before deployment of the device. Although several techniques were attempted, we were not able to decrease AI. Therefore, we did not deploy the device, and during the same procedure, surgical VSD closure was performed. Patients with severe valvular insufficiency during the procedure immediately underwent surgical VSD closure. Therefore, the incidence was relatively lower than that of transcatheter VSD closure, but there are no studies to support this hypothesis.

Device dislodgement: this problem may occur during or after the procedure and can appear as a significant shunt. Hong et al.^[5]^ reported its incidence as 2/1368 (0.14%), and Chen^[2]^ reported its incidence as 3/1033 (0.3%). The most important factor for the device dislodgement is underestimation of the defect size and lack of a suitable location for the device^[2]^. When we retrospectively analyzed our patient with device dislodgement (and surgical findings of this patient), we found that the tricuspid septal leaflet restricted the color Doppler flow to the defect and caused the defect to be underestimated. In addition, selecting a smaller device resulted in device dislodgement. Unfortunately, we realized the main reason for the problem the next day during surgery, when we observed the defect.

Arrhythmias: Chen et al.^[2]^ reported severe arrhythmias in 25/1033 (2.4%) patients. This number included 11 cases of cAVB and 14 cases of Mobitz type II atrioventricular (AV) block during and after the procedure. Xing et al.^[7]^ reported iRBBB in 11/393 patients (2.8%). The iRBBB resolved in 6 of the 11 patients, and the other 5 patients remained stable. In our series, the only arrhythmia was iRBBB. It was observed in two patients, and one of their iRBBB resolved during follow-up. 

cAVB: in our series, there was no AV block. This finding was due to the number of patients. The incidence of AV block is reported to be less than 1%, and our sample size was less than 100^[2,5]^. 

### Conversion to Conventional Repair

Fang et al.^[3]^ analyzed the factors for switching to conventional repair. In their series, residual shunts (8/340; 2.4%) and valvular insufficiency (5/340; 1.5%) were the main factors. When we reviewed the meta-analysis and large series reported by Hong et al.^[5]^, a total of 80/1368 (5.8%) patients were converted to conventional surgical repair. The reasons for conversion to conventional surgical repair included significant residual shunt (36.4%), mild to significant AI (35.2%), severe arrhythmia (11.4%), failure to establish a path (9.1%), and mild to significant TI (8.0%). Chen et al.^[2]^ reported conversion to conventional surgical repair in 57/1090 patients (5.2%) The reasons for conversion to conventional repair were generally the same as those reported in the published literature^[11,15,16]^. However, other than valvular insufficiency, stenosis (tricuspid valve stenosis or right ventricular outflow obstruction) could infrequently be a reason for conversion to conventional repair^[7,15]^. Device-dependent problems, such as abnormal device plasticity or device distortion, might occur^[7]^. In our series, the rate of conversion to conventional surgical repair was 2/59 (3.4%), and the indications were similar to those in the literature. In addition, one patient underwent conversion to surgical repair due to device dislodgement one day after the procedure. 

### Surgical Approach

This type of defect can be closed via mini-incision with surgery and during hybrid procedures; surgeons can use different types of mini-incisions. Surgery could be performed through a parasternal intercostal incision, 2-cm inferior median sternotomy, transverse sternal split, totally endoscopic surgery, right anterolateral thoracotomy, right parasternal incision (peratrial route), right submammary incision, left infra-axillary approach, right infra-axillary thoracotomy, or totally endoscopic surgery. In addition, hybrid periventricular VSD closure could be performed via some of these surgical incisions^[1,6,12,15,16]^. It is even reported without thoracotomy and direct puncture of the chest^[12]^. Our preference is a lower partial median sternotomy incision. If the device closure is unsuccessful, we can easily convert to conventional repair, which is performed by direct extension of the incision without another incision. 

### ICU Stay And Hospital Stay

This ICU stay (15 to 29 hours) and hospital stay (5.4 to 6.6 days) for this procedure were relatively shorter than those for surgical procedures^[2,6]^. This time difference was significantly higher than that for surgical VSD closure patients^[6]^. In our series, the mean ICU stay was 22±10 hours (range, 5-63 hours), which was similar to that in the literature. In contrast, the hospital stay was 9±4 days (range, 4-20 days) and was relatively longer than that in the literature. This difference is due to our department’s hospitalization regulations. Before 2019, to observe early postoperative arrhythmias, we did not discharge patients before 7 days after surgery, especially if the patients lived in rural areas. After 2019, we shortened this period up to 4 days. In addition, patients are required to wait for government approval for discharge, which requires two more days. 

### The Role of Periventricular VSD Closure in the Future

From our point of view, surgery is still the gold standard for VSD treatment with low mortality and morbidity rates. However, the hybrid procedure has additional advantages (which are mentioned above) and complication rates comparable to surgical and transcatheter methods. The future of periventricular VSD closure is promising for selected cases.

### Limitations

This was a retrospective, single-center study, with a limited number and wide age range of patients without a control group. The follow-up duration was limited, which may be important for the cAVB incidence.

## CONCLUSION

Our results show that the perventricular approach provides direct access and facilitates manipulation of the device position and orientation during device deployment. In addition, with the guidance of TEE, we can instantly evaluate major complications and simultaneously convert to surgical closure in cases with major complications. This procedure can be easily learned by surgeons and can be used in selected cases with promising results.

**Table:** 

Authors' roles & responsibilities	
LW	Substantial contributions to the conception or design of the work; or the acquisition, analysis or interpretation of data for the work; drafting the work or revising it critically for important intellectual content; final approval of the version to be published
ICT	Substantial contributions to the conception or design of the work; or the acquisition, analysis or interpretation of data for the work; drafting the work or revising it critically for important intellectual content; final approval of the version to be published
DTY	Substantial contributions to the conception or design of the work; or the acquisition, analysis or interpretation of data for the work; drafting the work or revising it critically for important intellectual content; final approval of the version to be published
XZ	Substantial contributions to the conception or design of the work; or the acquisition, analysis or interpretation of data for the work; drafting the work or revising it critically for important intellectual content; final approval of the version to be published
BL	Substantial contributions to the conception or design of the work; or the acquisition, analysis or interpretation of data for the work; final approval of the version to be published
DZ	Substantial contributions to the conception or design of the work; or the acquisition, analysis or interpretation of data for the work; final approval of the version to be published
GD	Substantial contributions to the conception or design of the work; or the acquisition, analysis or interpretation of data for the work; drafting the work or revising it critically for important intellectual content; final approval of the version to be published
HC	Substantial contributions to the conception or design of the work; or the acquisition, analysis or interpretation of data for the work; drafting the work or revising it critically for important intellectual content; final approval of the version to be published

## References

[1] Bacha E, Kalfa D (2014). Minimally invasive paediatric cardiac surgery. Nat Rev Cardiol.

[2] Chen Q, Hong ZN, Zhang GC, Chen LW, Zhang QL, Lin ZW (2018). Intraoperative device closure of isolated ventricular septal defects: experience on 1,090 cases. Ann Thorac Surg.

[3] Fang J, Xie S, Ma L, Yang C (2018). Anatomic and surgical factors affecting the switch from minimally invasive transthoracic occlusion to open surgery during ventricular septal defect repair. J Thorac Dis.

[4] Gupta A, Amin Z (2017). Popular hybrid congenital heart procedures without cardiopulmonary bypass. Front Surg.

[5] Hong ZN, Chen Q, Huang LQ, Cao H (2019). A meta-analysis of perventricular device closure of perimembranous ventricular septal defect. J Cardiothorac Surg.

[6] Hu Y, Li Z, Chen J, Li F, Shen C, Song Y (2015). Results of comparing transthoracic device closure and surgical repair with right infra-axillary thoracotomy for perimembranous ventricular septal defects. Interact Cardiovasc Thorac Surg.

[7] Xing Q, Pan S, An Q, Zhang Z, Li J, Li F (2010). Minimally invasive perventricular device closure of perimembranous ventricular septal defect without cardiopulmonary by-pass: multicenter experience and mid-term follow-up. J Thorac Cardiovasc Surg.

[8] Yin S, Zhu D, Lin K, An Q (2014). Perventricular device closure of congenital ventricular septal defects. J Card Surg.

[9] Tanidir IC, Baspinar O, Saygi M, Kervancioglu M, Guzeltas A, Odemis E (2020). Use of Lifetech(tm) Konar-MF, a device for both perimembranous and muscular ventricular septal defects: a multicentre study. Int J Cardiol.

[10] Chessa M, Butera G, Butera G, Chessa M, Eicken A, Thomson J (2015). Ventricular Septal Defects. Cardiac Catheterization for Congenital Heart Disease From Fetal Life to Adulthood.

[11] Fang GH, Chen Q, Hong ZN, Lin ZW, Zhang GC, Cao H (2018). The comparison of perventricular device closure with transcatheter device closure and the surgical repair via median sternotomy for perimembranous ventricular septal defect. Ann Thorac Cardiovasc Surg.

[12] Gan C, Peng L, Liang Z, Song H, Yang J, Ruan W (2017). Percutaneous perventricular device closure of ventricular septal defect.: from incision to pinhole. Ann Thorac Surg.

[13] Gray RG, Menon SC, Johnson JT, Armstrong AK, Bingler MA, Breinholt JP (2017). Acute and midterm results following perventricular device closure of muscular ventricular septal defects: a multicenter PICES investigation. Catheter Cardiovasc Interv.

[14] Ou-Yang WB, Wang SZ, Hu SS, Zhang FW, Zhang DW, Liu Y (2017). Perventricular device closure of perimembranous ventricular septal defect: effectiveness of symmetric and asymmetric occluders. Eur J Cardiothorac Surg.

[15] Garg P, Bishnoi AK, Patel K, Annanthnarayan C, Patel J, Talsariya M (2017). Transverse split sternotomy: a mini-invasive approach for repair of congenital cardiac defects. Innovations (Phila).

[16] Zhou S, Zhao L, Fan T, Li B, Liang W, Dong H (2017). Perventricular device closure of doubly committed sub-arterial ventricular septal defects via a left infra-axillary approach. J Card Surg.

